# Relative Quantitative Proteomic Analysis of *Brucella abortus* Reveals Metabolic Adaptation to Multiple Environmental Stresses

**DOI:** 10.3389/fmicb.2017.02347

**Published:** 2017-11-29

**Authors:** Xiaodong Zai, Qiaoling Yang, Ying Yin, Ruihua Li, Mengying Qian, Taoran Zhao, Yaohui Li, Jun Zhang, Ling Fu, Junjie Xu, Wei Chen

**Affiliations:** Laboratory of Vaccine and Antibody Engineering, Beijing Institute of Biotechnology, Beijing, China

**Keywords:** *Brucella abortus*, proteomic, label-free, environmental stress, differentially expressed protein, metabolic pathway

## Abstract

*Brucella* spp. are facultative intracellular pathogens that cause chronic brucellosis in humans and animals. The virulence of *Brucella* primarily depends on its successful survival and replication in host cells. During invasion of the host tissue, *Brucella* is simultaneously subjected to a variety of harsh conditions, including nutrient limitation, low pH, antimicrobial defenses, and extreme levels of reactive oxygen species (ROS) via the host immune response. This suggests that *Brucella* may be able to regulate its metabolic adaptation in response to the distinct stresses encountered during its intracellular infection of the host. An investigation into the differential proteome expression patterns of *Brucella* grown under the relevant stress conditions may contribute toward a better understanding of its pathogenesis and adaptive response. Here, we utilized a mass spectrometry-based label-free relative quantitative proteomics approach to investigate and compare global proteomic changes in *B. abortus* in response to eight different stress treatments. The 3 h short-term *in vitro* single-stress and multi-stress conditions mimicked the *in vivo* conditions of *B. abortus* under intracellular infection, with survival rates ranging from 3.17 to 73.17%. The proteomic analysis identified and quantified a total of 2,272 proteins and 74% of the theoretical proteome, thereby providing wide coverage of the *B. abortus* proteome. By including eight distinct growth conditions and comparing these with a control condition, we identified a total of 1,221 differentially expressed proteins (DEPs) that were significantly changed under the stress treatments. Pathway analysis revealed that most of the proteins were involved in oxidative phosphorylation, ABC transporters, two-component systems, biosynthesis of secondary metabolites, the citrate cycle, thiamine metabolism, and nitrogen metabolism; constituting major response mechanisms toward the reconstruction of cellular homeostasis and metabolic balance under stress. In conclusion, our results provide a better understanding of the global metabolic adaptations of *B. abortus* associated with distinct environmental stresses. The identification of proteins necessary for stress resistance is crucial toward elucidating the infectious process in order to control brucellosis, and may facilitate the discovery of novel therapeutic targets and effective vaccines.

## Introduction

*Brucella* (Brucellaceae) are gram-negative, facultative intracellular pathogens that cause brucellosis, which results in abortion and infertility in the natural host (Akpinar, 2016). Brucellosis is a major global zoonosis that infects approximately 500,000 people annually (Hasanjani Roushan and Ebrahimpour, 2015). *Brucella abortus, B. melitensis*, and *B. suis* are most pathogenic toward humans and have been listed as high priority biological agents (Doganay and Doganay, 2013). However, the pathogenic mechanisms of *Brucella* are currently not well understood. During invasion of the host tissue, the bacteria multiply inside phagocytic cells and eventually establish persistent infection and replication within the host (Ahmed et al., 2016). It appears that *Brucella* species do not depend on single discrete virulence factors such as cytolysins, capsules, exotoxins, secreted proteases, fimbriae, or phage-encoded toxins for their pathogenicity (He, 2012). Rather, their pathogenicity mainly depends on their capacity to survive and proliferate within host cells (Byndloss and Tsolis, 2016). During the invasion of host tissue, these bacteria are subjected to several severe stresses, including nutrient limitation, low pH, antimicrobial defenses, and extreme levels of reactive oxygen species (ROS) from the immune response of the host (Roop et al., 2009; Barbier et al., 2011; Olsen and Palmer, 2014). The pathogen may therefore be able to withstand the variety of stresses encountered during its intracellular infection (Lamontagne et al., 2009).

In recent years, proteomics has become an indispensable tool used to investigate the metabolic adaptation mechanisms of various organisms to multiple environmental stresses (Cash, 2011; Van Oudenhove and Devreese, 2013; Greco and Cristea, 2017). The assessment of differential proteome expression patterns of a pathogen under stress may contribute to a better understanding of pathogen adaptation and pathogenesis. Distinct environmental conditions can be simulated by *in vitro* models, in which bacterial cultures are exposed to different *in vivo*-mimicking conditions experienced in the cellular environment of the host. The metabolic adaptation of *Brucella* to specific stresses such as nutrient starvation, acidity, high temperature, or peroxide has been explored in previous studies (Teixeira-Gomes et al., 2000; Al-Dahouk et al., 2008, 2009, 2013). However, earlier proteomic approaches employed two-dimensional electrophoresis (2-D), and are therefore limited in their detection of alkaline and low-abundance proteins. Lamontagne et al. (2009) used an LC-MS approach to investigate and compare global proteomic changes in *B. abortus* at different times after infection *in vivo*, which provided insight into mechanisms utilized by *Brucella* to survive and proliferate within host cells. During the invasion of host tissue, *Brucella* is simultaneously subjected to a variety of harsh environments (Roop et al., 2009); however, previous proteomic studies failed to test a range of potential environmental stresses that the bacteria could be exposed to within the host.

In this study, we utilized a label-free relative quantitative proteomics approach to investigate and compare global proteomic changes in *B. abortus* in response to a variety of typical environmental stresses. A total of 2,272 proteins were identified and quantified, with significant changes observed in 1,221 under the multiple stress conditions tested. The differentially expressed proteins (DEPs) identified that were significantly changed under the stress treatments may provide novel insights into the global metabolic adaptations of *B. abortus* to multiple stresses. The identification of proteins necessary for stress resistance is crucial to elucidate the infection process and may facilitate the discovery of novel therapeutic targets and effective vaccines.

## Materials and methods

### *Brucella* strains and experimental design

The *B. abortus* 104-M strain was obtained from the Lanzhou Institute of Biological Products in China (Yu et al., 2015). The cells were subjected to a control treatment [grown on tryptic soy broth (TSB), condition #1] and eight different stress treatments as previously described, with some modifications (Teixeira-Gomes et al., 2000; Al-Dahouk et al., 2008, 2009, 2013; Lamontagne et al., 2010). These included: (i) seven single-stress conditions: #2 serum stress [addition of 10% serum (obtained from healthy volunteers after informed consent; stored at −20°C)]; #3 nutrient starvation stress (grown in Sauton's glycerol medium); #4 physical/chemical stress [grown in an acidic, high-temperature, hyperhaline, and high osmotic pressure condition (pH 4.5, 45°C, NaCl 1 mol/L final concentration, sorbitol 1 mol/L final concentration)]; #5 peroxide/nitric oxide stress (addition of 50 mM H_2_O_2_ and 5 mM DETA-NO, final concentrations); #6 oxygen deficiency stress (incubation sealed); #7 iron-limited stress [addition of 50 μM final concentration of the Fe^2+^-chelator 2,2′-dipyridyl (DIP; Sigma-Aldrich, Shanghai, China)]; #8 antibacterial stress (addition of polymyxin B to 100 μg/mL final concentration); and (ii) a multi-stress condition, consisting of a combination of conditions #2 to #8 (#9, see Table 1 for details).

**Table 1:** Survival of *B. abortus* under environmental stress.

**Group**	**Stress**	**Conditions**	**Cell survival rate (%)**
1	TSB (Control condition)	37°C, TSB, pH 7.6	100
2	Serum stress	10% serum	65.85
3	Nutrient starvation stress	Sauton's glycerol medium	5.85
4	Physical/chemical stress	45°C, TSB, pH 4.5, NaCl 1 mol/L, Sorbitol 1 mol/L	36.59
5	Peroxide/nitric oxide stress	H_2_O_2_ 50 mM, DETA-NO 5 mM	11.71
6	Oxygen deficiency stress	Incubation sealed	5.85
7	Iron-limited stress	Fe^2+^-chelator 2,2′-dipyridyl 50 μM	73.17
8	Antibacterial stress	Polymyxin B 100 μg/mL	24.39
9	Multi-stress	42°C, Sauton's glycerol medium, pH 5.5, NaCl 0.5 mol/L, Sorbitol 0.5 mol/L, 5% serum, H_2_O_2_ 25 mM, DETA-NO 2.5 mM, incubation sealed, Fe^2+^-chelator 2,2′-dipyridyl 25 μM, polymyxin B 50 μg/mL	3.17

The cells were first cultured in TSB with continuous shaking (200 rpm) at 37°C for approximately 24 h until mid-log phase (OD_600nm_ = 1.0). The cells were harvested by concentrating the solution, and were then resuspended in the eight different growth conditions listed above and incubated for 3 h in a shaking incubator. Cultures were then serially diluted and plated on tryptic soy agar to determine their viability post-challenge. The survival percentage of the 3 h post-stress challenge was calculated by dividing the number of colony-forming units obtained from each stress treatment to that obtained from the control treatment, multiplied by 100. The survival experiments were performed at least three times for each treatment. All experiments involving live *B. abortus* 104-M were conducted in BSL-2 labs in line with health and safety guidelines.

### Protein sample preparation and proteolytic digestion

The protein samples were prepared as described previously (Zai et al., 2017). Briefly, cells cultured in each condition were harvested by centrifugation (7,000 × g for 15 min), and then washed three times with phosphate-buffered saline (PBS). The bacterial cells were resuspended in lysis buffer and disrupted by ultrasonication (25% amplitude, 15 min at 0°C). The resultant suspension was centrifuged (40,000 × g for 30 min) and the protein concentrations in the collected supernatants were measured using a BCA (bicinchoninic acid) protein assay kit (Thermo Fisher Scientific, Waltham, USA). The cell protein extracts were reduced in 1 mM dithiothreitol (25°C for 1 h) and then alkylated in 5.5 mM iodoacetamide (25°C for 1 h, in the dark). Sequencing-grade trypsin (Promega) was added to a final ratio of 1:50 (V:V) and the proteins were digested in solution overnight at 37°C.

### Liquid chromatography coupled to tandem mass spectrometry (LC-MS/MS)

All experiments were performed on an LTQ Q-Exactive HF mass spectrometer (Thermo Scientific, USA) coupled online with a nano-HPLC (Ultimate 3000, Thermo Scientific) (Scheltema et al., 2014). The peptides were loaded onto a trap column (C18, 3 μm particles, 100 μm × 2 cm) and separated on EASY-Spray columns (C18, 1.9 μm particles, 15 μm × 12 cm) with trapping at a flow rate of 600 nL/min (Kentache et al., 2017). The mobile phase A was 0.1% formic acid in water and the mobile phase B was 0.1% formic acid in acetonitrile. The peptides were eluted using a gradient (6–95% mobile phase B) during a 195-min LC run and then sprayed directly into the MS instrument.

The mass spectrometer was operated using the data-dependent top-15 method with automatic switching between MS and MS/MS scans (Kalayou et al., 2016). Full MS scans were acquired at a resolution of 120,000, with an automatic gain control target value of 3 × 10^6^ ions or maximum injection time of 80 ms within the scan range 300–1,400 m/z. Peptide fragmentation was performed by higher energy collision dissociation (HCD) with the normalized collision energy set to 27 (Tutturen et al., 2014). The 15 highest-intensity ions were then selected for the collision-induced fragmentation at a resolution *R* = 15,000, an automatic gain control target value of 5 × 10^4^ ions, or maximum fragment accumulation time of 45 ms. After the fragmentation event, dynamic exclusion of precursor ion masses for 12 s was used to avoid the repeated fragmentation of peaks. We excluded precursor ions with single, unassigned, or ≥ seven charge states from the fragmentation selection.

### Protein identification and label-free quantification

Protein identification was performed by submitting raw data files to Proteome Discoverer software (Thermo Scientific, USA, v. 1.2). The MS/MS searches were performed using the SEQUEST (v. 28) algorithm against a database constructed from the UniProt entries for *B. abortus* 104-M (taxonomy: 1210454), which contained 3,072 protein sequences. The search parameters included specific digestion with trypsin with up to two missed cleavages allowed; carbamidomethylation (C) on cysteine was set as a fixed modification; and oxidation (M) on methylene and acetyl (protein-N term) on asparagine & glutamine were applied as variable modifications. The initial allowed mass deviation of the precursor ion was set to 15 ppm, and the allowed value for the fragment mass was set to 0.02 Da. Protein identifications that contained at least two identified peptides were accepted with a false discovery rate (FDR) less than 1.0% (Zai et al., 2017).

For protein quantification, a label-free experiment was performed as previously described (Pettersen et al., 2016; Schmidt et al., 2016). Briefly, raw data were imported into Proteome Discover 1.4 following the MS analysis. Protein abundance of was calculated on the basis of label-free quantitation intensity [LFQ]. For comparison, the protein abundance in the control group was set as a reference and the protein abundance in the other eight stress groups were aligned. Proteins identified in at least two out of nine groups were considered for label-free quantification. Those proteins exhibiting fold change >1.5 (*P*-value ≤ 0.05) between the treatment and the control were deemed up-regulated or down-regulated respectively. The MS proteomics data have been deposited in the ProteomeXchange Consortium via the jPOSTrepo (Japan ProteOme STandard Repository) with the data set identifier PXD007548 (Vizcaino et al., 2014; Okuda et al., 2017).

### Bioinformatics analyses

The calculation of protein molecular masses, pI, and peptide grand average of hydropathicity (GRAVY) values were carried out using the ProtParam tool from the ExPASy toolbox (Gasteiger et al., 2003). Protein transmembrane helices were predicted using TMHMM 2.0 (Krogh et al., 2001). The protein functions were assigned by the Clusters of Orthologous Groups (COG) database (Galperin et al., 2015). The pathways of proteins were analyzed using the Kyoto Encyclopedia of Genes and Genomes (KEGG) database (Kanehisa et al., 2017). Possible interactions between identified proteins were tested using the STRING tool (Search Tool for the Retrieval of Interacting Genes/Proteins) (Szklarczyk et al., 2017). Heatmaps of the proteins were generated using the versatile matrix visualization and analysis software Morpheus, available from the Broad Institute.

### Experimental design and statistical rationale

The proteomes of bacteria grown under the nine groups were investigated. For each condition, two biological replicates and two technical replicates were sampled, resulting in a total of four samples per condition for LC-MS/MS. Protein identifications should contain at least two identified peptides with a FDR less than 1.0%. Significant label-free changes in proteins were determined using the statistical analysis-based variance (ANOVA) test, which was performed on the protein LFQ values. Additionally, principal coordinate analysis (PCoA) was used to visualize the correlations among the nine groups. The Pearson's *r* correlation coefficient between the nine groups was also visualized by means of a correlation matrix. The expression patterns of the proteins grown under the nine conditions were presented in a heatmap with hierarchical clustering performed using a Euclidean distance metric and the average linkage method (Pettersen et al., 2016).

## Results

### Experimental design

**Figure 1:**
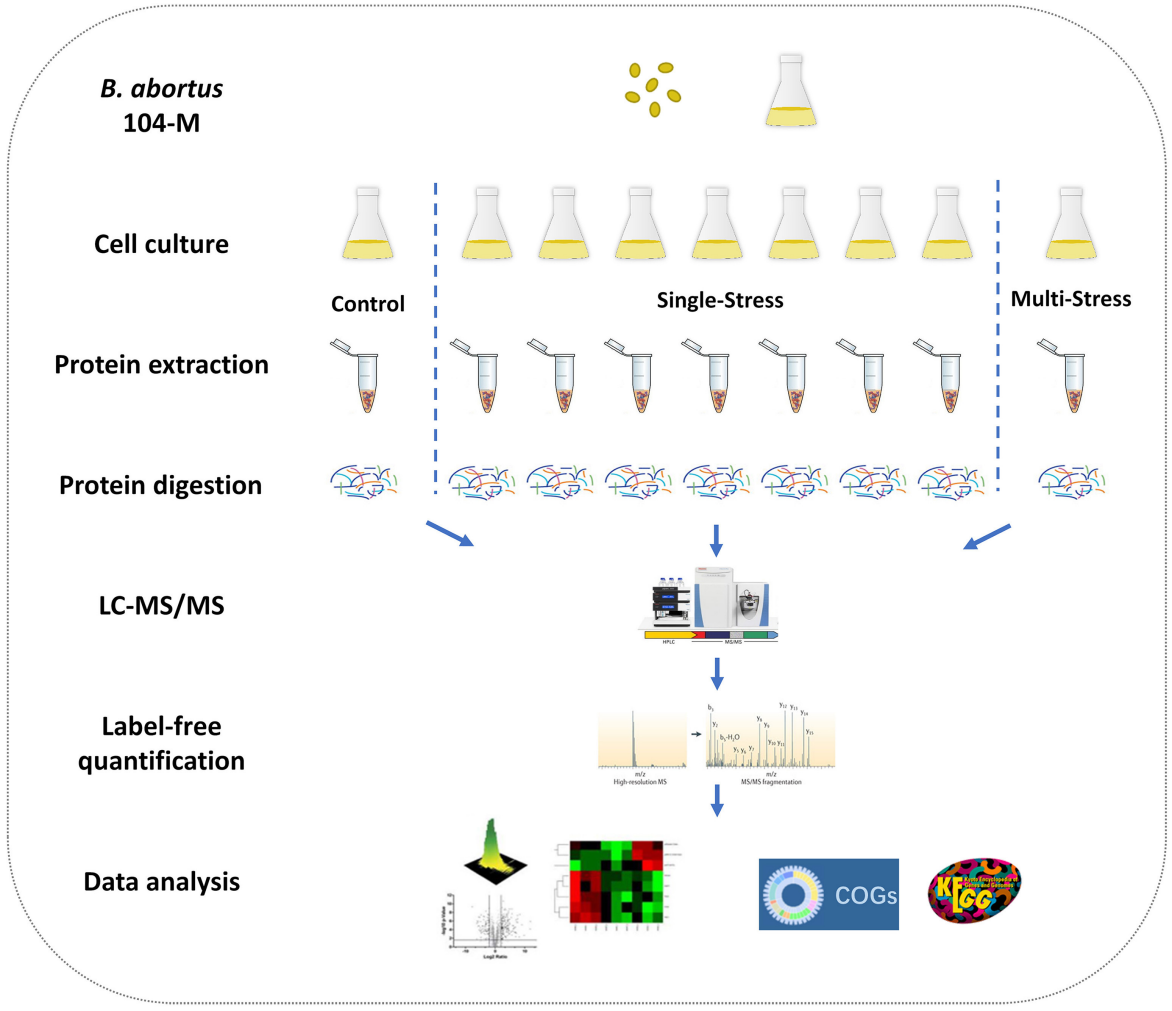
Experimental design of the quantitative proteomics of *Brucella abortus* under environmental stress. A label-free relative quantitative proteomics approach was utilized to investigate and compare the global proteomic changes of *B. abortus* in response to a variety of distinct stresses including a control condition, seven single-stress conditions, and a multi-stress condition. Protein samples were prepared and proteolytic-digested using trypsin enzymes. Peptides were analyzed on a Q-Exactive HF MS coupled online with a nano-HPLC. The identified proteins were quantified using a label-free approach and further functionally analyzed using the COG and KEGG databases.

*Brucella* spp. are facultative intracellular bacteria. During the invasion of host tissue, *Brucella* are subjected to various harsh environmental conditions including nutrient limitation, low pH, antimicrobial defenses, and extreme levels of ROS (Roop et al., 2009; Barbier et al., 2011; Olsen and Palmer, 2014). Correspondingly, *Brucella* is well-equipped from both a physiological and metabolic perspective to adopt to environmental stresses (Roop et al., 2009). Analysis of the differential proteome expression patterns of *Brucella* under stress should improve our understanding regarding its adaptation and pathogenesis. Thus, we chose a multiple-environmental-stress strategy to reveal the global metabolic adaptations of *B. abortus* to intravacuolar environmental conditions. These conditions included: (i) a control condition (growth on TSB, condition #1); (ii) seven single-stress conditions: (conditions #2–8) and (iii) a multi-stress condition (#9, see Table 1 for details). The multi-stress condition constituted a combination of each single-stress condition, and may more realistically simulate the conditions experienced by *Brucella* during infection of the host. We investigated the survival rate and differential protein expression of *B. abortus* in the single-stress and multi-stress conditions compared with the control condition (Figure 1). The survival rates of *B. abortus* under the different stress conditions ranged from 3.17 to 73.17%. Nutrient starvation, physical/chemical starvation, peroxide/NO starvation, and anaerobic starvation resulted in low survival rates (Table 1). The multi-stress condition resulted in the lowest survival rates, and may constitute a more accurate reflection of the *in vivo* conditions of *B. abortus* under intracellular infection.

### Coverage of the *B. abortus* proteome

**Figure 2:**
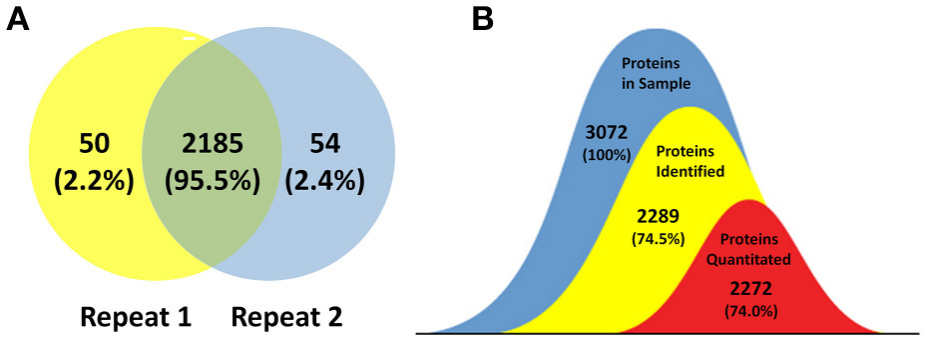
Coverage of the *B. abortus* proteome by high-resolution mass spectrometry. **(A)** Venn plot of protein identification overlaps among two independent biological experiments. The number of proteins identified in the *B. abortus* proteome were 2,289, with 2,235 and 2,239 in the respective experiments. **(B)** The number of proteins identified in at least two out of nine groups and considered for label-free quantification was 2,272. The coverage of proteins identified and proteins quantitated in this study was 74.5 and 74%, respectively.

The goal of this study was to achieve substantial coverage of the *B. abortus* proteome under *in vitro* stress conditions. Accordingly, we applied a proteomics approach on whole cell lysates prepared from *B. abortus* grown in the nine groups described above. LC-MS/MS analysis of the resulting peptide mixtures generated 1.1 million spectra. The acquired raw MS data files were then analyzed and the spectral files matched to 27,076 unique peptides with an FDR of 1.0%. We mapped the unique peptides to the *B. abortus* 104-M UniProt database (3,072 protein sequences), and only proteins that were identified by at least two unique peptides were confirmed (Zai et al., 2017). A total of 2,289 proteins were identified by two repeats, which represents approximately 74.5% coverage of the predicted proteome (Figure 2A). Proteins identified in at least two out of nine groups (2,272) were considered for label-free quantification (Figure 2B). The distribution of the identified proteins with respect to pI, molecular weight, hydrophobicity, and transmembrane regions was consistent with the annotated proteins (Figure S1). Among the 2,289 identified proteins, 1,570 were annotated in the COG database. Almost all of the pivotal categories for *Brucella* were identified, suggesting good coverage and representation of the genomic content of *B. abortus* by the proteome. Table S1 lists all identified proteins with their accession numbers and calculated score.

### Quantification analysis of proteins under environmental stress

**Figure 3:**
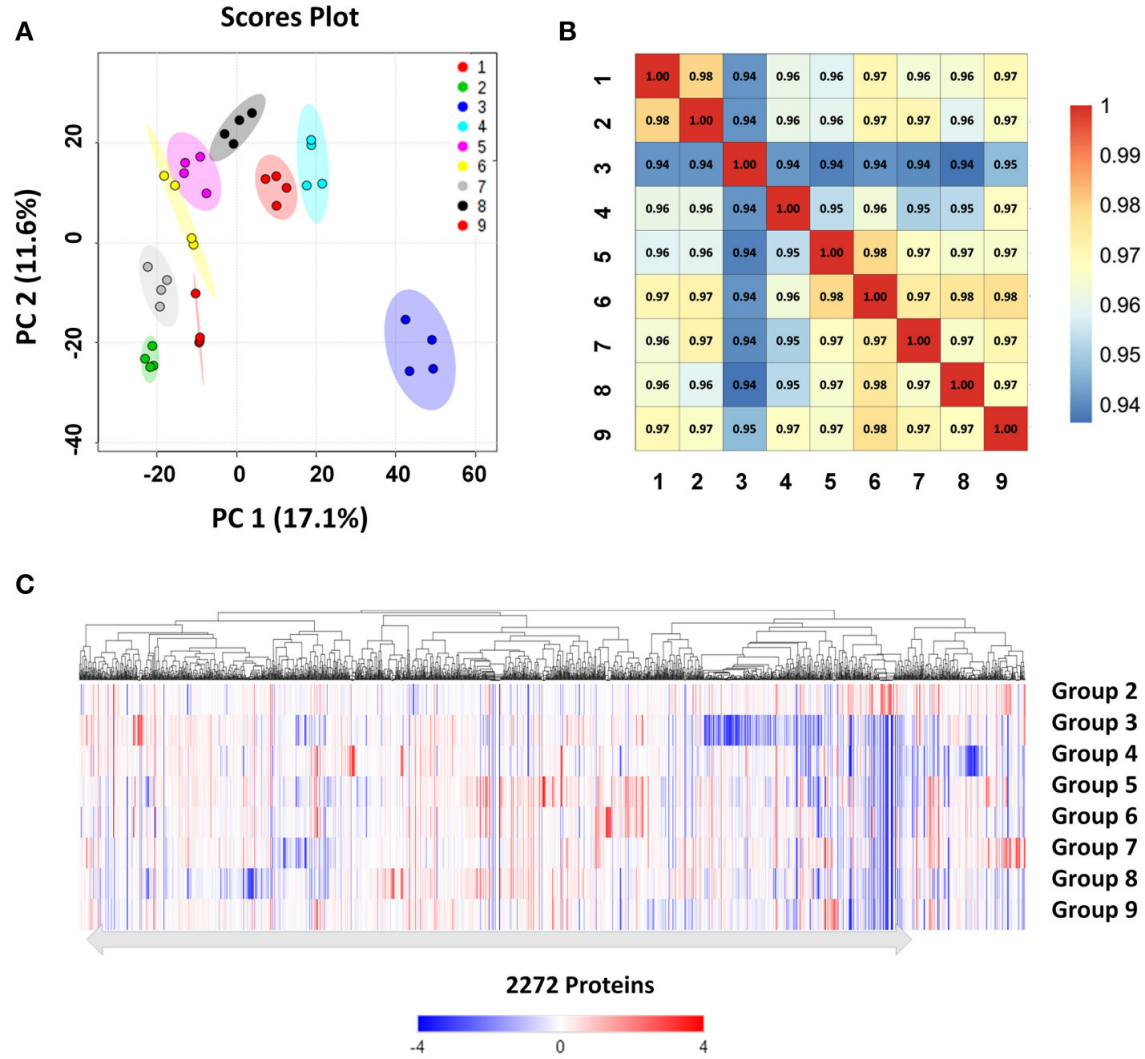
Clustering and correlation of quantified proteins of *B. abortus* under environmental stress. **(A)** PCoA comparing the level of variance among the biological and technical replicates in the nine growth conditions for all the 2,272 proteins obtained after one-way ANOVA. Each dot represents an independent biological replicate. **(B)** Pearson's correlation coefficients of the protein intensities in all nine groups compared against each other. **(C)** Heatmap and hierarchical clustering of quantified proteins from cells under stress treatments. Hierarchical clustering was performed based on all quantified proteins. Relative protein expression under stress conditions was compared to the control condition.

We used high-resolution MS to quantitatively describe the protein profiles of *B. abortus* under stress conditions, and compared them with the expressed proteome in the control. Protein quantification allowed the characterization of proteins that were differentially expressed across the nine groups. Here, we obtained LFQ intensities for 2,272 quantified proteins. Their quantitative levels covered a 5-log_10_ dynamic range. The density plot of the log2 ratio between the stress group and control group closely matched a normal distribution, which indicated that the experimental procedure was performed without systematic bias toward the different samples. PCoA analysis indicated good homogeneity of the biological replicates and was able to discriminate nine distinct protein populations (Figure 3A). The Pearson's correlation coefficient *r* varied between 0.95 and 0.99, indicating that there were differences in the protein levels between the different stress conditions (Figure 3B).

We next explored differences between the protein profiles of *B. abortus* under the various stress conditions. Hierarchical clustering was performed based on the LFQ intensities of the 2,272 proteins quantified in this study, and indicated distinguishable protein expression profiles between the nine different groups (Figure 3C). For each growth condition, a range of 1,967–2,109 proteins per group were identified (Table 2). About 87.6% of the detected proteins were common to all groups. Several of the identified proteins demonstrated a significant difference in abundance in stress compared to the control condition. The study identified DEPs in each stress condition whose quantitative levels varied by more than 1.5 fold from the respective LFQ intensity means in the control, i.e., 213, 413, 311, 451, 314, 357, 401, and 306 proteins in the eight stress conditions, respectively (Figure S2). Table S2 lists all the DEPs along with their accession numbers and LFQ intensities for each condition.

**Table 2:** Overview of *B. abortus* proteins identified under environmental stress.

**Group**	**Stress**	**Identified proteins**	**Unique A^a^**	**Unique B^b^**	**Common^c^**	**Up^d^**	**Down^e^**
1	TSB (Control)	2,091	–	–	–	–	–
2	Serum stress	2,109	67	49	2,042	125	88
3	Nutrient starvation stress	1,967	49	173	1,918	139	274
4	Physical/chemical stress	2,001	46	136	1,955	120	191
5	Peroxide/NO stress	2,064	72	99	1,992	235	216
6	Oxygen deficiency stress	2,079	73	85	2,006	165	149
7	Iron-limited stress	2,085	68	74	2,017	169	188
8	Antibacterial stress	2,009	52	134	1,957	146	255
9	Multi-stress	2,046	58	103	1,988	116	190

a *Proteins unique to the stress condition*.

b *Proteins unique to the control*.

c *The common proteins identified in both the control and stress conditions*.

d *The up-regulated proteins under stress conditions*.

e *The down-regulated proteins under stress conditions*.

### Metabolic pathway analysis of DEPs under each environmental stress condition

To understand the functional classification and metabolic pathways that were involved in the environmental stress response, the DEPs in each stress condition were functionally analyzed. All of the enriched KEGG pathways of *B. abortus* in response to each stress treatment are listed in Table 3. The results suggested that the metabolic adaptations of *B. abortus* to each environmental stress condition varied.

**Table 3:** KEGG enrichment analysis of DEPs in response to each stress treatment.

**Group**	**Stress**	**Pathway-ID**	**Description**	**Genes**	***P*-value**
2	Serum stress	ko01053	Biosynthesis of siderophore group nonribosomal peptides	5	0.0000683
		ko02010	ABC transporters	24	0.0056025
		ko00072	Synthesis and degradation of ketone bodies	3	0.0099555
		ko02040	Flagellar assembly	3	0.0099555
		ko00730	Thiamine metabolism	3	0.0608771
		ko02020	Two-component system	10	0.0702431
3	Nutrient starvation stress	ko02020	Two-component system	22	0.0061642
		ko00564	Glycerophospholipid metabolism	8	0.0065323
		ko02010	ABC transporters	44	0.0081475
		ko00910	Nitrogen metabolism	9	0.0120856
		ko00860	Porphyrin and chlorophyll metabolism	14	0.0258088
		ko01053	Biosynthesis of siderophore group nonribosomal peptides	4	0.0297269
		ko00790	Folate biosynthesis	7	0.0791646
		ko00072	Synthesis and degradation of ketone bodies	3	0.087634
4	Physical/chemical stress	ko02010	ABC transporters	35	0.000864
		ko00730	Thiamine metabolism	5	0.0065975
		ko01053	Biosynthesis of siderophore group nonribosomal peptides	4	0.0067706
		ko00564	Glycerophospholipid metabolism	6	0.0136601
		ko02020	Two-component system	15	0.0233687
		ko00072	Synthesis and degradation of ketone bodies	3	0.0297309
		ko00910	Nitrogen metabolism	6	0.0491114
5	Peroxide/nitric oxide stress	ko00910	Nitrogen metabolism	9	0.0041133
		ko02010	ABC transporters	38	0.0133396
		ko01053	Biosynthesis of siderophore group nonribosomal peptides	4	0.0170841
		ko00730	Thiamine metabolism	5	0.0196635
		ko02020	Two-component system	18	0.0242233
		ko00860	Porphyrin and chlorophyll metabolism	12	0.0415006
		ko00564	Glycerophospholipid metabolism	6	0.0440453
		ko01220	Degradation of aromatic compounds	5	0.0732277
6	Oxygen deficiency stress	ko02020	Two-component system	16	0.005989
		ko02040	Flagellar assembly	3	0.025681
		ko00564	Glycerophospholipid metabolism	5	0.0441204
		ko02024	Quorum sensing	15	0.0521694
		ko02010	ABC transporters	27	0.0600535
		ko00350	Tyrosine metabolism	4	0.0887595
		ko00920	Sulfur metabolism	5	0.0942084
7	Iron-limited stress	ko00190	Oxidative phosphorylation	29	1.32E-12
		ko02020	Two-component system	24	0.0000285
		ko01053	Biosynthesis of siderophore group nonribosomal peptides	6	0.0000445
		ko00910	Nitrogen metabolism	11	0.0000831
		ko00020	Citrate cycle (TCA cycle)	12	0.0005147
		ko01120	Microbial metabolism in diverse environments	43	0.0275001
		ko00072	Synthesis and degradation of ketone bodies	3	0.0502816
		ko00860	Porphyrin and chlorophyll metabolism	11	0.0627104
		ko00650	Butanoate metabolism	7	0.0673582
		ko02010	ABC transporters	33	0.0682408
8	Antibacterial stress	ko02020	Two-component system	24	0.0000861
		ko00910	Nitrogen metabolism	11	0.0001522
		ko00190	Oxidative phosphorylation	16	0.006429
		ko01053	Biosynthesis of siderophore group nonribosomal peptides	4	0.0173993
		ko03410	Base excision repair	5	0.0200875
		ko02010	ABC transporters	37	0.0246314
		ko01220	Degradation of aromatic compounds	5	0.0745956
		ko00130	Ubiquinone and other terpenoid-quinone biosynthesis	4	0.0879378
9	Multi-stress	ko02020	Two-component system	18	0.0019099
		ko00910	Nitrogen metabolism	8	0.0037586
		ko00730	Thiamine metabolism	5	0.007187
		ko01053	Biosynthesis of siderophore group nonribosomal peptides	4	0.0072753
		ko02010	ABC transporters	30	0.0313728
		ko03410	Base excision repair	4	0.0411957

*Brucella* can persist for several weeks in the blood of an intraperitoneally-infected host (Vitry et al., 2014). Considering the role that serum stimulation has in intraperitoneally-infected hosts of *Brucella*, serum stress was chosen as single-stress condition #2. In this treatment, we discovered 125 and 88 up-regulated and down-regulated proteins, respectively, compared with the control. As shown in Figure S3A, the main enriched KEGG pathways for regulated proteins included biosynthesis of siderophore group non-ribosomal peptides, ABC transporters, synthesis and degradation of ketone bodies, thiamine metabolism, and two-component system. This suggests that *Brucella* may enhance its iron acquisition through the regulation of ABC transporters and biosynthesis of siderophores in order to adapt to the serum stress.

During the invasion of host tissue, a major hurdle in the infection of a host cell by *Brucella* is the lack of nutrients within the phagosome (Essenberg et al., 2002; Hanna et al., 2013; Barbier et al., 2017). Considering the role that nutrient starvation has in the intracellular replication of *Brucella*, nutrient stress was set as single-stress condition #3. Of the DEPs detected under this condition, 139 and 274 proteins were respectively up-regulated or down-regulated compared with the control. The primary enriched KEGG pathways for regulated proteins included two-component system, glycerophospholipid metabolism, ABC transporters, nitrogen metabolism, and porphyrin and chlorophyll metabolism (Figure S3B). This implies that *Brucella* may decrease its energy usage and secondary metabolite biosynthesis through the regulation of glycerophospholipid metabolism, nitrogen metabolism, and porphyrin and chlorophyll metabolism in response to nutrient starvation stress.

One major mechanism of *Brucella* pathogenesis is the capacity to survive in the acidic, high-temperature, hyperhaline, and high osmotic pressure environment inside macrophages (Detilleux et al., 1991; Liu et al., 2015). Given that physical/chemical stimulation influences the intracellular replication of *Brucella*, physical/chemical stress was set as single-stress condition #4. We discovered 120 and 191 up-regulated and down-regulated proteins present in the physical/chemical stress condition compared with the control. ABC transporters, thiamine metabolism, biosynthesis of siderophore group nonribosomal peptides, glycerophospholipid metabolism, and two-component system constituted the main enriched KEGG pathways (Figure S3C). *Brucella* may therefore decrease its energy usage and secondary metabolite biosynthesis through the regulation of glycerophospholipid metabolism and thiamine metabolism in order to the physical/chemical stress.

The exogenous production of ROS such as O^2−^ and H_2_O_2_ by the host immune system has also been shown to be important for the survival of *Brucella* (Jimenez De Bagues et al., 2007). Nitric oxide produced by the macrophages were also crucial for phagocytes to control the intracellular replication of *Brucella* (Roop et al., 2009; Ronneau et al., 2016). Peroxide/nitric oxide stress was therefore set as single-stress condition #5. Of the DEPs detected in this treatment, 235 and 216 proteins were up-regulated or down-regulated respectively compared with the control. The main enriched KEGG pathways for regulated proteins included nitrogen metabolism, ABC transporters, biosynthesis of siderophore group nonribosomal peptides, thiamine metabolism, and two-component system (Figure S3D). The result suggests that *Brucella* may decrease its amino acid usage and secondary metabolite biosynthesis through the regulation of nitrogen metabolism and thiamine metabolism, meanwhile enhancing its iron acquisition through regulation of the two-component system and biosynthesis of siderophores in response to peroxide/NO stress.

The presence of oxygen has positive effects on the levels of proteins that are functional in aerobic respiration and purine metabolism, while low access to oxygen results in the induction of enzymes of mixed-acid fermentation and gluconate metabolism (James et al., 1995). Considering the role that oxygen deficiency plays in the intracellular replication of *Brucella*, oxygen deficiency stress was set as single-stress condition #6. In this treatment we discovered that 165 and 149 proteins were up-regulated or down-regulated respectively compared with the control. The main enriched KEGG pathways for the regulated proteins were two-component system, flagellar assembly, glycerophospholipid metabolism, quorum sensing, and ABC transporters (Figure S3E). Thus, *Brucella* may decrease its energy usage and virulence through the regulation of glycerophospholipid metabolism, two-component system, flagellar assembly and quorum sensing to manage oxygen deficiency stress.

Iron is an essential element for *Brucella* and is pivotal in host-pathogen interactions (Eskra et al., 2012; Roop, 2012). In the host, free iron levels are extremely low, resulting in iron limitation being a crucial stress (Braun, 2001). Iron-limitation stress was thus set as single-stress condition #7. We found that 169 and 188 proteins were respectively up-regulated or down-regulated in the iron-limited stress condition compared with the control. As indicated in Figure S3F, the main enriched KEGG pathways included oxidative phosphorylation, two-component system, biosynthesis of siderophore group nonribosomal peptides, nitrogen metabolism, and citrate cycle (TCA cycle). This result suggests that *Brucella* may decrease its energy usage through the regulation of oxidative phosphorylation, nitrogen metabolism and the TCA cycle, while enhancing its iron acquisition through the regulation of siderophore biosynthesis to adapt to the stress resulting from limited iron.

Antimicrobial peptides can limit the colonization of bacterial during infection in the innate defense, and therefore it is likely that *Brucella* encounters antimicrobial peptides within host microenvironments during infection (Martinez De Tejada et al., 1995). These peptides may be involved in environmental signaling that triggers changes in bacterial gene expression. Considering the role that antimicrobial peptide stimulation has in the intracellular replication of *Brucella*, antibacterial stress was set as single-stress condition #8. Of the DEPs in this treatment, 146 and 255 proteins were up-regulated or down-regulated respectively in comparison with the control. The main enriched KEGG pathways included two-component system, nitrogen metabolism, oxidative phosphorylation, biosynthesis of siderophore group nonribosomal peptides, and base excision repair (Figure S3G). This suggests that *Brucella* may decrease its energy usage through the regulation of oxidative phosphorylation and nitrogen metabolism, while enhancing its antimicrobial peptide resistance through regulation of the two-component system and base excision repair in response to antibacterial stress.

During the invasion of host tissue, *Brucella* experiences several stresses simultaneously, including nutrient limitation, low pH, antimicrobial defenses, and extreme ROS levels from the host immune response. Thus, the combination of stress conditions 2 to 8 was set as the multi-stress condition #9 that may better simulate the environments that *Brucella* may occur during host infection. We discovered that 116 and 190 proteins were respectively up-regulated or down-regulated in the multi-stress condition compared with the control. The primary enriched KEGG pathways included two-component system, nitrogen metabolism, thiamine metabolism, biosynthesis of siderophore group nonribosomal peptides, and ABC transporters (Figure S3H). *Brucella* may decrease its energy usage and secondary metabolite biosynthesis via the regulation of nitrogen metabolism and thiamine metabolism, while enhancing its iron acquisition and antimicrobial peptide resistance by regulating the two-component system and siderophore biosynthesis in order to cope with multiple stresses.

Compared with the single-stress condition, the enriched pathways of *B. abortus* in response to multi-stress conditions were more extensive and covered the primary enriched pathways of each single-stress condition. For example, the two-component system pathway enriched in the multi-stress condition was also enriched in all eight of the single-stress conditions, indicating its significance in the metabolic adaptation to multiple environmental stresses. The nitrogen metabolism pathway was enriched in the multi-stress condition and also in numerous single-stress conditions, including nutrient starvation stress, physical/chemical stress, peroxide/nitric oxide stress, iron-limited stress, and antibacterial stress. The DEPs included in this enriched pathway were similar in their regulatory patterns in both the single-stress and multi-stress treatments. The results suggested that the metabolic adaptations of *B. abortus* to multiple stresses constitute the synthesis of each environmental stress condition, and that the multiple stress treatment may better simulate the metabolic adaptations of *Brucella* during host infection.

### Metabolic pathway analysis of DEPs under various stress conditions

**Figure 4:**
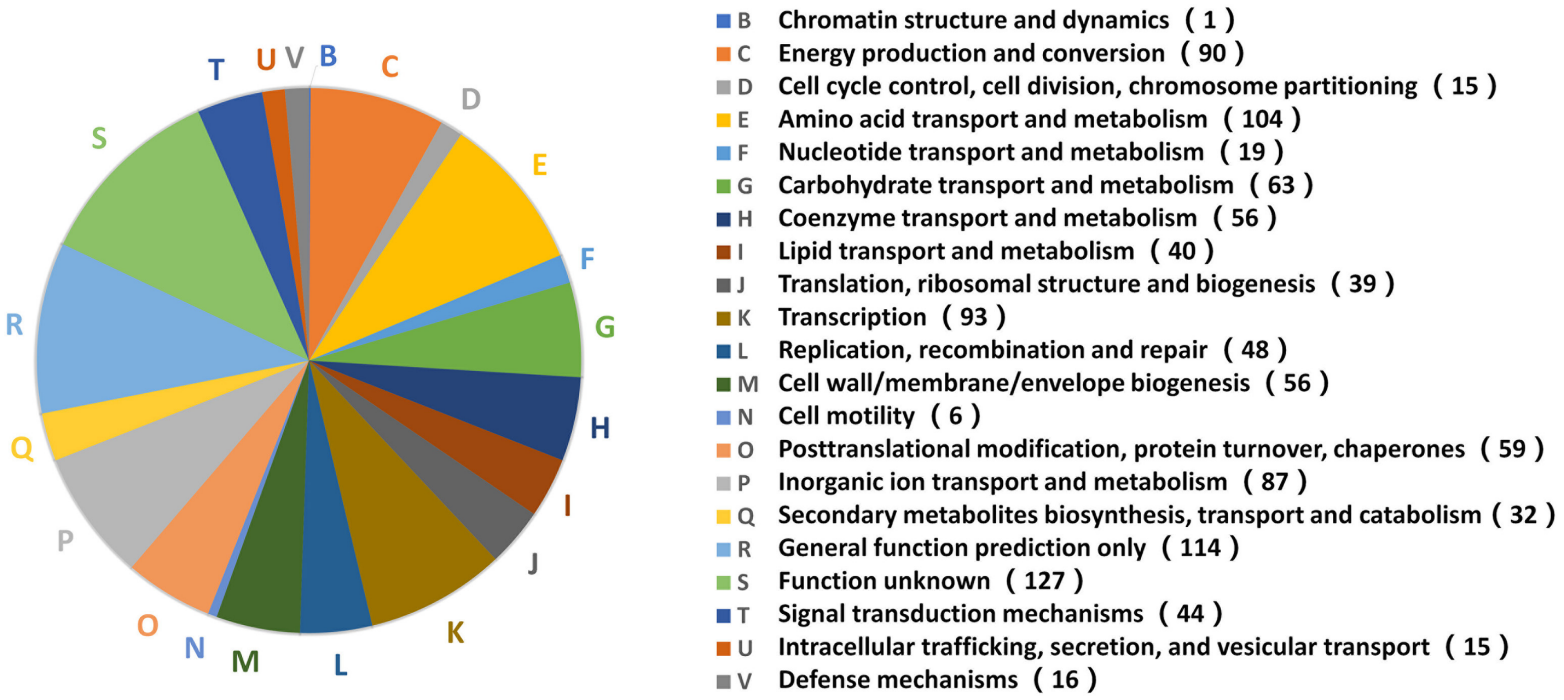
Distribution of 1,221 DEPs in the stress treatments according to COG functions. A phylogenetic classification of DEPs in response to environmental stress was accomplished using the clusters of COGs. Letters displayed on the pie chart represent individual COGs with the numbers of proteins listed in brackets afterwards.

The functional analysis above indicated that *B. abortus* exhibited different metabolic adaptations to each environmental stress condition. To better understand the primary metabolic adaptations of *B. abortus* to various stress conditions, we summed all the DEPs in each stress condition, resulting in a total of 1,221 proteins that were differentially expressed in at least one stress condition compared with the control. The protein functions of all 1,221 DEPs in the eight stress conditions were assigned by the COG database. We identified 20 different COG phylogenetic protein groups (Figure 4), with the highest represented subsets in category E (amino acid transport and metabolism, 104 proteins), category K (transcription, 93 proteins), category C (energy production and conversion, 90 proteins), category P (inorganic ion transport and metabolism, 87 proteins), and category G (carbohydrate transport and metabolism, 63 proteins). Furthermore, 114 proteins were assigned only a putative function (category R), while 127 proteins remained without an allocated biological role (category S). The COG functional analysis suggested that energy usage-related categories (E, C, and G) and the iron acquisition-related category (P) were involved in the main metabolic adaptations of *B. abortus* to various stress conditions.

**Figure 5:**
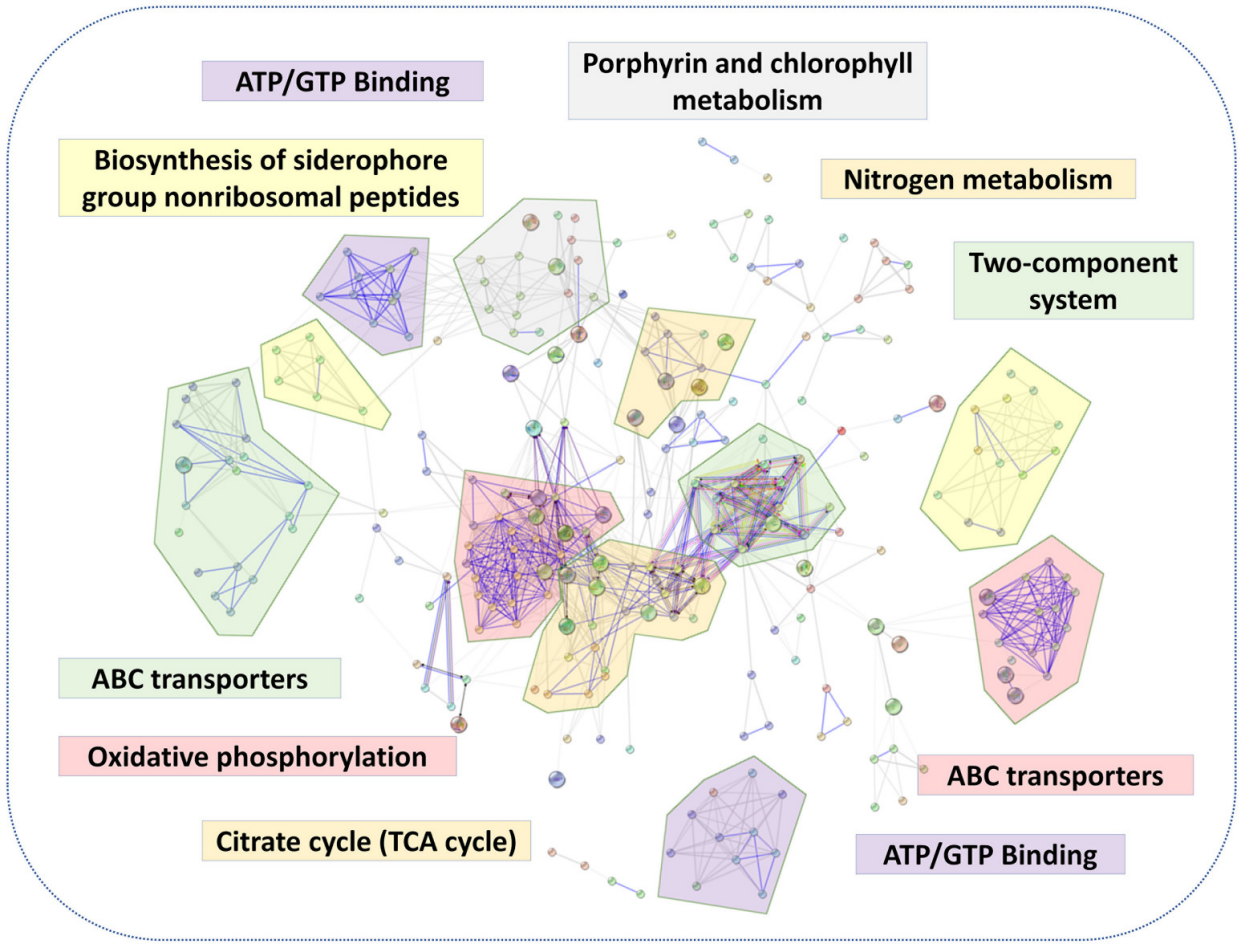
The main metabolic changes of *B. abortus* in response to stress treatments. Possible interactions between DEPs under the stress treatments were tested using the STRING software tool. The network utilized *1st shell* that contained 442 nodes and 1,904 edges with confidence score (0.7). KEGG pathway analyses of DEPs indicated major metabolic adaptation mechanisms of *B. abortus* in response to multiple environmental stresses.

A putative protein-protein interaction network was then constructed from all high-confidence *B. abortus* protein interaction pairs that were matched with the 1,221 DEPs using the STRING tool (Szklarczyk et al., 2015). The KEGG pathway analysis showed a significant enrichment of DEPs (*P*-value ≤ 0.05). All 1,221 DEPs in the eight stress conditions were primarily enriched in oxidative phosphorylation, ABC transporters, two-component systems, biosynthesis of secondary metabolites, porphyrin and chlorophyll metabolism, glycerol phospholipid metabolism, the TCA cycle, thiamine metabolism, nitrogen metabolism, or were associated with carbon metabolism (Figure 5). The KEGG pathway analysis suggested that the energy usage-related pathways and regulatory mechanism pathways constituted the primary pathways involved in the metabolic adaptation of *B. abortus* to various stress conditions. Table S3 lists the DEPs that were related to the main metabolic changes of *B. abortus* in response to stress treatments.

### Main metabolic changes in response to environmental stress

**Figure 6:**
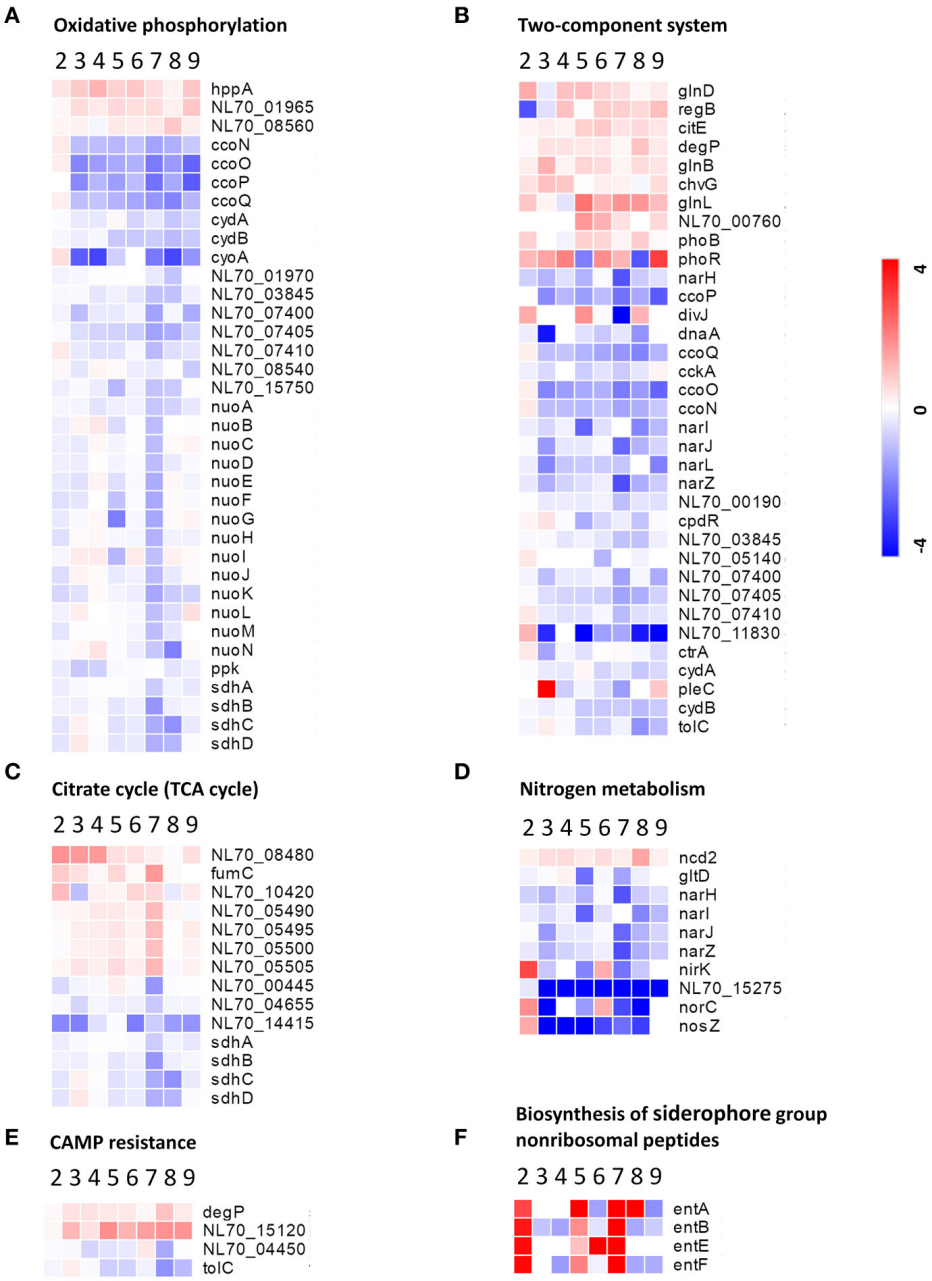
Heatmap of DEPs related to the main metabolic changes of *B. abortus* in response to stress treatments. Fold change of proteins related to **(A)** oxidative phosphorylation, **(B)** two-component systems, **(C)** the citric acid cycle, **(D)** nitrogen metabolism, **(E)** cationic antimicrobial peptide resistance, and **(F)** biosynthesis of siderophore group nonribosomal peptides. The heatmap is shown in matrix format with rows representing the individual proteins and columns representing each stress condition. The red and blue colors reflect high and low fold changes, respectively.

Oxidative phosphorylation is the process in which ATP is formed through using enzymes to oxidize nutrients (Dimroth et al., 2000). TCA cycle is a series of chemical reactions that produces ATP through the oxidation of acetyl-CoA. In this study, several proteins involved in oxidative phosphorylation and the TCA cycle, such as those associated with the NADH dehydrogenase (NDH) family, succinate dehydrogenase (SDH), and cytochrome *c* oxidase CcO, were abundantly down-regulated in response to both the single-stress and multiple-stress treatments (Figures 6A,C). A major hurdle in the infection of a host cell by *Brucella* is the lack of nutrients within the phagosome (Barbier et al., 2017). These results suggest that immediately after phagocytosis, *Brucella* reduces energy metabolism via the TCA cycle as the available extracellular nutrients decrease.

Two-component systems are adapted to respond to a wide variety of stress, including nutrients, quorum signals, antibiotics, temperature, pH, and so forth (Mascher et al., 2006). In this study, several proteins involved in two component system-controlled metabolic pathways, such as chromosomal replication initiator protein dnaA and nitrate reductase Nar, were decreased in abundance. Two-component system sensor histidine kinase PhoR/GlnL, hybrid sensor histidine kinase/response regulator ChvG, and transcriptional regulator RegB were up-regulated in response to several single-stress and multiple environmental stresses (Figure 6B). During the invasion of host tissue, *Brucella* must survive under several severe stresses through generating a suitable adaptive response to various signals (Viadas et al., 2010). Here, we found that the two-component system proteins were regulated in response to stress, which is essential for the persistence of *Brucella* within stressed environments in the host organism.

Cationic antimicrobial peptides (CAMPs) are crucial for the host defense against invasive bacterial infection (Alegado and Tan, 2008). The resistance of pathogenic bacteria toward antimicrobial peptides may also account for their virulence. In this study, several proteins involved in CAMPs resistance, such as the serine peptidase DegP and Hemolysin D (NL70_15120), were increased in abundance in our study. Transporter TolC and N-acetylmuramoyl-L-alanine amidase (NL70_04450) were down-regulated in response to several single environmental stresses, especially antibacterial stress (Figure 6E). These results indicated that the adopted mechanism of *Brucella* to resist host antimicrobials is important for persistent infection.

Siderophores are an important group of structurally diverse natural products that chelate iron and are important in the acquisition of the essential trace element iron by most microorganisms (Miethke and Marahiel, 2007). In the host, free iron levels are extremely low as the metal is largely bound to proteins. To overcome iron limitation, some bacteria and fungi produce siderophores. In this study, several proteins involved in the biosynthesis of siderophore group nonribosomal peptides, such as enterobactin biosynthetic enzymes EntA/B/E/F, were found to be up-regulated particularly in response to serum stress and iron-limitation stress (Figure 6F). These results indicated that *Brucella* has evolved strategies to overcome iron limitation and to compete with the iron sequestration immune mechanisms of the host.

We also observed specific variations in the amounts of multiple proteins involved in nitrogen metabolism, thiamine metabolism, and purine metabolism. In this study, nitrate reductase (Nar), nitrite reductase (NirK), and thiamine pyrophosphokinase (ThiN/E/G), which play essential roles in amino acid metabolism, and several enzymes participating in glycine, serine, and threonine metabolism, were relatively significantly differentially expressed in the different conditions (Figure 6D). Serine peptidase HtrA and heat shock protein Hsp20 were observed to increase considerably under both the single-stress and multiple-stress treatments. The Cu-Zu superoxide dismutase SodC was found to increase during oxidative stress and multiple-stress treatments, but remained unchanged under the physical/chemical stress conditions of heat shock or acidic pH. Other proteins such as exopolyphosphatase (NL70_13665), ribonuclease H (RnhA), single-stranded DNA exonuclease (RecJ), and urea amidohydrolase (UreA/B/C) were also reduced in at least one of the stress conditions in our study.

## Discussion

The virulence of *Brucella* strains mainly depends on their capacity to survive and proliferate within host cells (Kohler et al., 2002). During the invasion of host tissue, *Brucella* is able to withstand the environmental stresses encountered and establish and maintain persistent intracellular residence (Roop et al., 2009). However, the mechanisms used by *Brucella* in intracellular infection are not fully understood. The aim of this work was to elucidate the regulatory processes of *Brucella* that enable survival under extreme stress by mirroring the possible living conditions of the bacteria in the host environment.

Multiple approaches have been used to investigate the proteomes of bacterial pathogens (Schmidt and Volker, 2011; Semanjski and Macek, 2016). Since bacteria are much smaller than mammalian host cells, the detection of bacterial proteins is difficult due to interference from the large excess of host proteins present. As a result of these challenges, the majority of bacterial proteomic datasets are still obtained from *in vitro* experiments (Cash, 2011). The main advantage of *in vitro* systems is the ability to implement a simple experimental design in a controlled manner using defined media and conditions. Although the *in vivo* approach in cultured cell lines reflects the conditions in infected hosts more closely than *in vitro* cultures, and reveals pathogenesis-related determinants present throughout the course of infection, it only partially describes the actual infection state due to the artificial conditions of the cell culture. Thus, *in vitro* approaches significantly contribute toward our understanding of the physiology of pathogenic bacteria and assist in identifying novel virulence factors that may represent potential biomarkers or drug targets. However, previous *in vitro* studies did not test the range of possible environment stresses that *Brucella* may be exposed to within the host. During the invasion of host tissue, *Brucella* is simultaneously subjected to a variety of harsh environments. Distinct environmental conditions can be simulated in *in vitro* models in which bacterial cultures are exposed to different *in vivo*-mimicking conditions similar to the cellular environment of the host. We therefore chose a multiple-environmental-stress strategy in order to reveal the global metabolic adaptations of *B. abortus* to intravacuolar environmental conditions.

We used a label-free proteomics approach to quantitatively elucidate the protein profiles of *B. abortus* under conditions of stress, and compared them with the expressed proteome in the control sample. The *in vitro* single-stress and multi-stress conditions simulated the *in vivo* conditions of *B. abortus* under intracellular infection, with survival rates ranging from 3.17 to 73.17%. The results suggested that all of the single-stress conditions constituted harsh environments for *B. abortus*. The multi-stress condition resulted in the lowest survival rates, and may constitute a more accurate reflection of the *in vivo* conditions of *B. abortus* under intracellular infection. During the invasion of host tissue, *Brucella* is subjected to a harsh environment that results in the vast majority of the cells being killed within the macrophages (Di-Russo Case and Samuel, 2016). The *in vitro* stress treatments utilized in this study correspond well with the *in vivo* conditions of *Brucella* under intracellular infection.

The proteomic analysis identified and quantified a total of 2,272 proteins and 74% of the theoretical proteome, which has provided wide coverage of the *B. abortus* proteome. By replicating eight typical stress environments *in vitro*, we were able to investigate the influence of various stresses on the detected proteomes. The results indicated that there were different metabolic adaptations to different environmental stresses. The COG functional analysis of the 1,221 DEPs showed that energy usage-related categories (E, C, and G) and the iron acquisition-related category (P) were involved in the main metabolic adaptations of B. abortus to various stress conditions. While the KEGG pathway analyses revealed that the majority of pathways were involved in oxidative phosphorylation, ABC transporters, two-component systems, biosynthesis of secondary metabolites, the citrate cycle, thiamine metabolism, and nitrogen metabolism; all representing major response mechanisms involved in the maintenance of cellular homeostasis and metabolic balance under stress. The multi-stress treatment was a combination of each single-stress, and may better reflect the metabolic response of *Brucella* under intracellular infection. Most of the regulated proteins in the multi-stress treatment were associated with oxidative phosphorylation, the citrate cycle, nitrogen metabolism, and biosynthesis of secondary metabolites, suggesting that *Brucella* may decrease the oxidation of nutrients, amino acid use and reduce secondary metabolite biosynthesis in order to adapt to the intracellular environment.

Teixeira-Gomes et al. (2000) studied the differences in protein synthesis patterns in *B. melitensis* 16M in response to heat, oxidative, and acidic pH stresses using a 2-D approach. The 19 resulting DEPs suggested that *B. melitensis* invoked an adaptive response to stress conditions. Al-Dahouk et al. characterized the proteome of *B. suis* at the late stage of *in vitro* infection, oxygen deficiency and long-term nutrient starvation using a 2-D approach, respectively (Al-Dahouk et al., 2008, 2009, 2013). The resulting 168, 37, and 30 DEPs indicated the regulatory mechanisms that reducing processes participating in energy, protein, and nucleic acid metabolism. Lamontagne et al. (2009) characterized the proteome of *B. abortus* strain 2308 and attenuated strain 19 that were infected into macrophages. The comparative analysis suggested that the *B. abortus* initially reduced the majority of biosynthesis and altered its respiration, but these adaptations were reversed later in the infection process.

Our observations are consistent with previous studies that suggest that *Brucella* may regulate its metabolism by decreasing its energy usage and secondary metabolite biosynthesis, while enhancing its iron acquisition and two-component system to cope with the intracellular environment (Kohler et al., 2002). In addition, we also discovered that some unique pathway categories and regulated genes play key roles in stress resistance, like thiamine metabolism and purine metabolism, thereby further elucidating the metabolic adaptation of *Brucella* to specific stressors. Furthermore, on comparison with the intracellular lifecycle of *Mycobacterium tuberculosis*, we discovered that the latter adapted to the intracellular environment by producing several key virulence factors which also appear on *Brucella* (Weiss and Schaible, 2015). For example, the virulence regulator PhoR in the two-component system plays a major role in *M. tuberculosis* pathogenicity and is also regulated in *Brucella* in response to stress (Ryndak et al., 2008; Broset et al., 2015). These findings may facilitate a better understanding of the metabolic adaptations of intracellular pathogens during their infection lifecycle.

The quantitative data obtained here are the most comprehensive to date that might capture the integral proteome profiling by *B. abortus* at a specific point in time. However, there may be some limitations regarding our experimental system. We used *in vitro* stress conditions to mimic the *in vivo* condition of *B. abortus* under intracellular infection by culturing the cells in a rich medium (TSB) until mid-log phase, and then transferring them to the stress treatments for 3 h. TSB is a standard culture medium that is routinely used as the control condition in proteomic analyses of *Brucella* under stress (Teixeira-Gomes et al., 2000). Compared with the extracellular lifecycle (represented here by culture on TSB), *Brucella* is subjected to severe nutrient limitation when invading the host tissue (represented by the treatments). Additionally, the selected 3 h treatment duration used constituted a short-term stress treatment that approximates the preliminary stage required for the survival of *Brucella* in the host cells (Lamontagne et al., 2009). We suspect that gradual metabolic changes may occur in *Brucella* after 3 h. However, we recommend that other conditions using different media and stress durations are tested in future research.

In conclusion, we have utilized a label-free relative quantitative proteomics approach to describe the protein profiles of *B. abortus* under different stress conditions. Under the multi-stress treatment, *B. abortus* experienced greater survival pressure in an environment that better imitates the intracellular environment of the host. Our results revealed differences in protein expression between the different stress treatments, providing new insight into the metabolic pathway of the response of *B. abortus* to multiple environmental stresses. Further studies into the proteins required for stress resistance under multiple environmental stresses are warranted to elucidate metabolic adaptation in *Brucella*. Continued efforts to elucidate the manner in which *Brucella* has adapted to its intracellular niche should provide valuable information for the discovery of novel therapeutic targets and effective vaccines in order to control brucellosis.

## Author contributions

XZ, JX, and WC conceived and designed the experiments; XZ, QY, RL and LF performed the experiments; XZ, MQ and YY analyzed the data; YL, TZ and YY contributed to the reagents; XZ and JX wrote the paper. All authors read and approved the finalized manuscript.

### Conflict of interest statement

The authors declare that the research was conducted in the absence of any commercial or financial relationships that could be construed as a potential conflict of interest.

## Abbreviations

2-D: two-dimensional electrophoresis
CAMPs: cationic antimicrobial peptides
DEP: differentially expressed protein
LC-MS/MS: liquid chromatography coupled to tandem mass spectrometry
LTQ: Linear trap quadrupole
FDR: False discovery rate
COG: Clusters of orthologous groups
KEGG: Kyoto Encyclopedia of Genes and Genomes
STRING: Search Tool for the Retrieval of Interacting Genes/Proteins
LFQ: label-free quantitation
PCoA: principal coordinate analysis.
