# Supplementary material for: Relative Quantitative Proteomic Analysis of Brucella abortus Reveals Metabolic Adaptation to Multiple Environmental Stresses
Source: Front Microbiol. 2017 Nov 29;8:2347. doi: 10.3389/fmicb.2017.02347 (PMC5712581; doi:10.3389/fmicb.2017.02347)
Supplement: Supplementary file 1 [file DataSheet1.PDF]

Supplementary Material

Relative Quantitative Proteomic Analysis of *Brucella abortus* Reveals Metabolic Adaptation to Multiple Environmental Stresses

Xiaodong Zai, Qiaoling Yang, Ying Yin, Ruihua Li, Mengying Qian, Taoran Zhao, Yaohui Li, Jun Zhang, Ling Fu, Junjie Xu*, Wei Chen*

*** Correspondence:** Junjie Xu, xujunjie@sina.com; Wei Chen, cw0226@foxmail.com

1 Supplementary Figures and Tables

1.1 Supplementary Figures

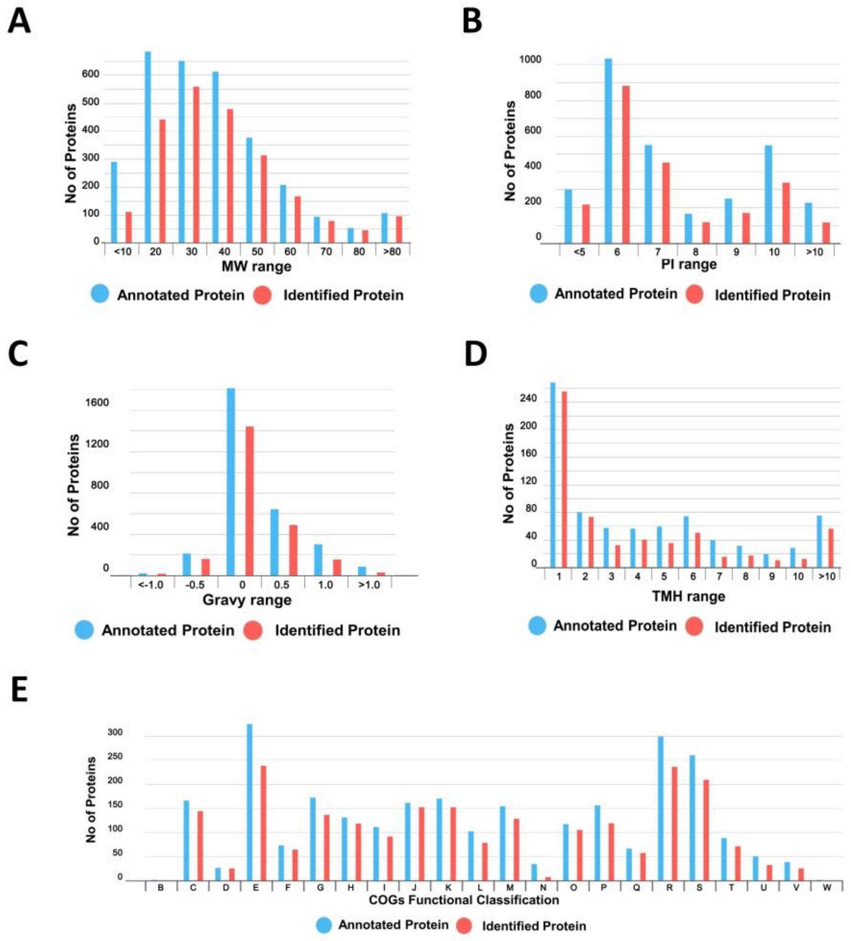

Supplementary Figure 1. Analysis of identified proteins of the *B. abortus* proteome in this study. The relative distribution of all proteins predicted by genome annotation in this study (shown in blue). All proteins could be identified using this proteome database (shown in red), according to (A) protein MW, (B) protein PI, (C) number of transmembrane proteins, (D) protein GRAVY, and (E) COG functional categories.

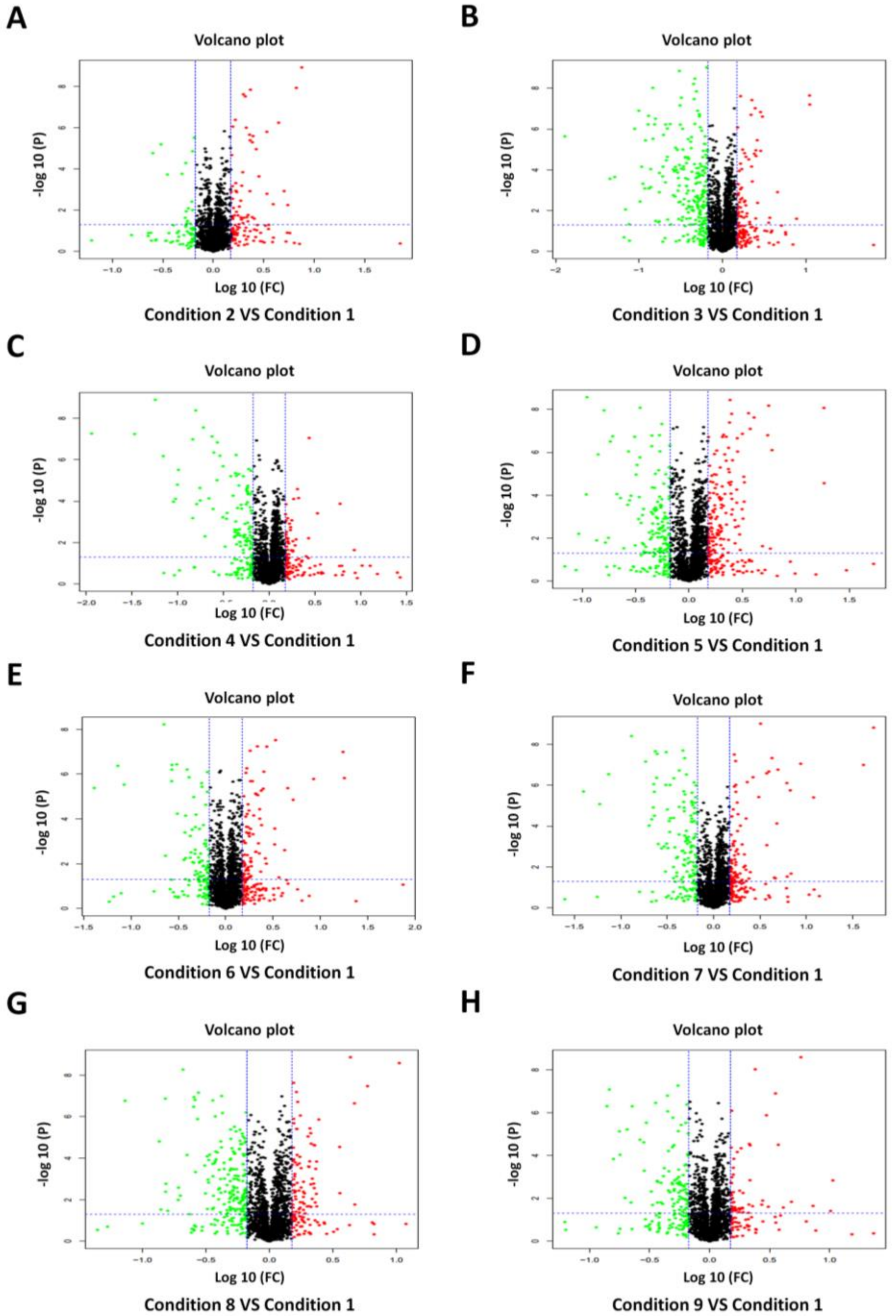

Supplementary Figure 2. Volcano plot of differentially expressed proteins between stress treatment and control. DEPs with quantitative levels that varied 1.5-fold from the respective LFQ intensity means in the control were identified under each of the seven single-stress conditions and the multi-stress condition: #2 serum stress (A); #3 nutrient starvation stress (B); #4 physical/chemical stress (C); #5 peroxide/nitric oxide stress (D); #6 oxygen deficiency stress (E); #7 iron-limited stress (F); #8 antibacterial stress (G); and a multi-stress condition #9 (H) respectively. The red/green color coding indicates up-regulation and down-regulation respectively. Proteins with no statistical significance are represented in black.

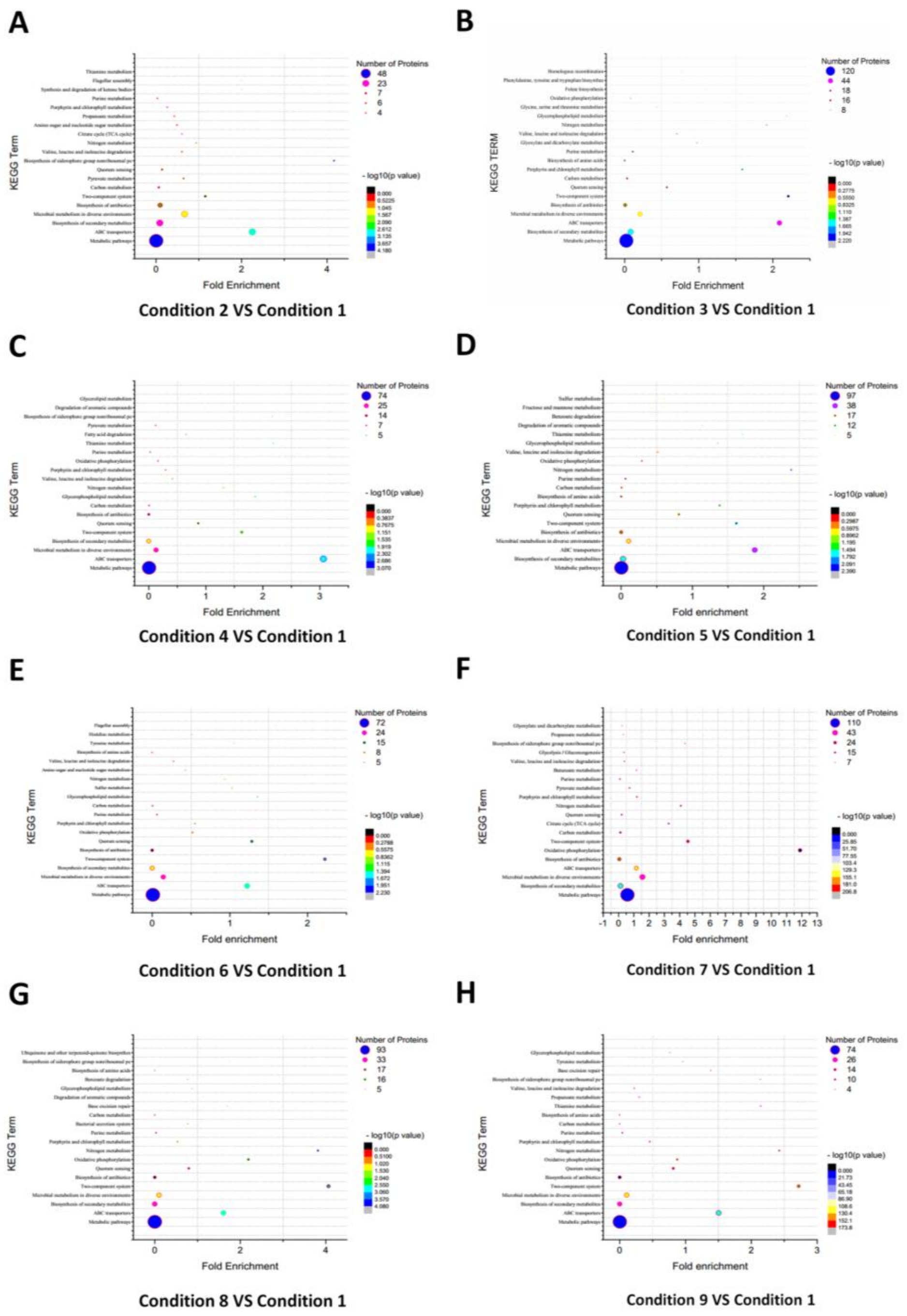

Supplementary Figure 3. KEGG enrichment analysis of DEPs in response to each stress treatment. To analyze the functional classification and metabolic pathways that were involved in the responses to the stress treatments, the DEPs in each condition including seven single-stress conditions: #2 serum stress (A); #3 nutrient starvation stress (B); #4 physical/chemical stress (C); #5 peroxide/nitric oxide stress (D); #6 oxygen deficiency stress (E); #7 iron-limited stress (F); #8 antibacterial stress (G); and a multi-stress condition #9 (H) were further analyzed using the KEGG database.

1.2 Supplementary Tables

Table S1: List of all the identified proteins in *B. abortus*

Table S2: List of all DEPs in *B. abortus*

Table S3: List of DEPs related to the main metabolic changes in *B. abortus*
